# Supplementary material for: Initial evaluation of nighttime restlessness in a naturally occurring canine model of osteoarthritis pain
Source: PeerJ. 2015 Feb 17;3:e772. doi: 10.7717/peerj.772 (PMC4340376; doi:10.7717/peerj.772)
Supplement: Appendix S1 — Sleep and Night Time Restlessness Evaluation Score (SNoRE) clinical metrology instrument used in Part B of this study. [file peerj-03-772-s001.docx]

**Sleep and Night time Restlessness Evaluation (SNoRE) score**

1. Over the last 7 days, what has been your dog’s ability to sleep without moving or getting up (restfull sleep)? Fill in the oval next to the number that best describes your dog’s ability to sleep:

Never moves Constant moving / never still

🔿1 🔿2 🔿3 🔿4 🔿5 🔿6 🔿7 🔿8 🔿9 🔿10

2. Fill in the oval next to the one number that best describes your dog’s sleep over the last 7 days

Never twitches Constant twitching

🔿1 🔿2 🔿3 🔿4 🔿5 🔿6 🔿7 🔿8 🔿9 🔿10

3. Fill in the oval next to the one number that best describes your dog’s sleep over the last 7 days

Never dreams Constant dreaming

🔿1 🔿2 🔿3 🔿4 🔿5 🔿6 🔿7 🔿8 🔿9 🔿10

4. Fill in the oval next to the one number that best describes your dog’s sleep over the last 7 days

Never shifts position Constantly shifting position

🔿1 🔿2 🔿3 🔿4 🔿5 🔿6 🔿7 🔿8 🔿9 🔿10

5. Fill in the oval next to the one number that best describes your dog’s sleep over the last 7 days

Never vocalizes Constantly vocalizing

🔿1 🔿2 🔿3 🔿4 🔿5 🔿6 🔿7 🔿8 🔿9 🔿10

6. Fill in the oval next to the one number that best describes your dog’s sleep over the last 7 days

Never paces Constantly pacing

🔿1 🔿2 🔿3 🔿4 🔿5 🔿6 🔿7 🔿8 🔿9 🔿10
